# Grading of minor salivary gland immuno-histopathology post-allogenic hematopoietic cell transplantation

**DOI:** 10.1016/j.heliyon.2023.e15517

**Published:** 2023-04-15

**Authors:** V. Tollemar, H. Arvidsson, H. Häbel, N. Tudzarovski, K. Garming Legert, K. Le Blanc, G. Warfvinge, R.V. Sugars

**Affiliations:** a Division of Oral Diagnostics and Rehabilitation, Department of Dental Medicine, Karolinska Institutet, Stockholm, Sweden; b Medical Statistics Unit, Department of Learning, Informatics, Management and Ethics, Karolinska Institutet, Stockholm, Sweden; c Division of Clinical Immunology and Transfusion Medicine, Department of Laboratory Medicine, Karolinska Institutet, Stockholm, Sweden; d Center of Allogeneic Stem Cell Transplantation and Cellular Therapy (CAST), Karolinska University Hospital Huddinge, Stockholm, Sweden; e Department of Oral Pathology, Faculty of Odontology, Malmö University, Malmö, Sweden

**Keywords:** Oral cGVHD, Histopathology, Grading, Large cohort, Hematopoietic cell transplantation

## Abstract

The oral cavity commonly displays mucosal lichenoid lesions and salivary gland dysfunction, which are considered different chronic Graft-versus-Host Disease (cGVHD) pathophysiology’s. However, diagnostics of salivary gland (sg-)cGVHD are limited. The objectives of the current study are to evaluate the minor salivary gland (MSG) histo-immunopathological profiles post allogenic hematopoietic cell transplantation based on sg-cGVHD criteria. **Design**: Histopathology was characterized according to two published grading strategies. Firstly, the National Institute of Health (NIH) assessed peri-ductal/acinar infiltration, exocytosis, damage, and fibrosis, and a points-based grading scheme was established (0–16 points, Grade (G) 0 to IV). Second, a modified Sjögren’s Syndrome focus-score with parenchymal damage was also adapted, (0–10 points, Score 0 to 2). 146 MSG biopsies from 79 patients were compared, using the histopathological specific criteria for sg-cGVHD pathology. Quantitative immunohistochemistry for T-cells (CD4, CD8), B-cells (CD19, CD20), monocytic cells (CD68) and dendritic cells (CD1a) were also assessed. **Results:** The large-scale cohort validated the use of both grading schemes. GIII-GIV and score 2 signified a histopathological diagnosis of “likely” sg-cGVHD. Immunopathological severity was associated with increased T-cells (CD4 and CD8) and monocytic (CD68) infiltrate, with minimal involvement of B-cells (CD19 and CD20), and Langerhans cells (CD1a). **Conclusions:** Both schemes were verified as being suitable for histological grading to improve assessment and diagnosis of sg-cGVHD. The NIH cGVHD grading appears to be more beneficial for research purposes, including final diagnostics of “no/inactive”, “possible” or “likely” cGVHD. The study highlights the intricacies of sg-cGVHD pathology; and the need for standardized assessment to improve patient management associated to sg-cGVHD.

## 1 Introduction

Allogenic hematopoietic cell transplantation (HCT) is a potential curative treatment for patients with various hematological disorders. However, donor cell engraftment elevates the risk of alloreactive acute (a) or chronic (c) Graft-versus-Host Disease (GVHD) [1]. cGVHD has a prevalence of 30–50% and can in the worst-case lead to significant morbidity and mortality [1,2]. cGVHD incidence is increasing in line with growing numbers of allogenic HCTs and with improved long-term survival [3]. Multiple organs might display cGVHD manifestations, but the oral cavity is one of the most prominent sites (45–83%) [4].

Salivary gland (sg)-cGVHD remains without any specific diagnostic criteria [5]. Mucoceles are distinctive for active oral cGVHD but might occur due to mucosal or salivary gland inflammation [5,6]. Xerostomia is also distinctive and has a reported prevalence of 60–77% in larger cGVHD cohorts [5,[7], [8], [9], [10]]. Unstimulated saliva flow ≤0.2 ml/min has been used as cut-off for dysfunctional salivary glands in patients with established cGVHD but is only reported with a prevalence of 11–27% [8,11]. However, the specificity of these clinical features is considered as relatively low, since subjective xerostomia and objective hyposalivation do not always cohere and can be influenced by confounding factors, such as medication and transplant-related issues [8,12,13]. Others have drawn on experience from similar disorders, such as Sjögren’s Syndrome (SS). SS-patients are diagnosed based on ≤0.1 ml/min unstimulated whole saliva, a Schirmer’s test showing ≤5mm/5min, ocular staining score of ≥5, labial MSG biopsy with a focus score of ≥1/4 mm^2^ and autoantibodies against SS-related antigen A [14].

Prior to the first National Institutes of Health (NIH) cGVHD Consensus Development Project Pathology Working Group, grading of minor salivary gland (MSG) cGVHD histopathology reporting was combined with mucosal features [[15], [16], [17], [18]]. However, increasing evidence suggests the need to distinguish between sg- and mucosal (om)-cGVHD pathophysiology’s [8,11]. The NIH cGVHD Pathology Group generated the resource document (https://www.astct.org/archive/practice-resources/nih-chronic-gvhd-consensus-project) that was validated in 2013, but to date no reports have used these features to conduct severity grading for guided diagnostics [9,18,19]. Lymphocytic inflammation and exocytosis around intralobular ducts and acini, with subsequent damage and fibrosis are specific features for sg-cGVHD. However, the infiltrate could be mixed as plasmocytic infiltrates might also be present [9,18,19]. Damage to intralobular ducts included vacuolization, nuclei dropout, dyspolarity and apoptosis [18,19]. Acinar apoptotic degeneration progresses with ductal metaplasia and reduced periodic acid Schiff (PAS) positive mucopolysaccharide stain [9,12,18,19]. However, tissue destruction could also be attributed to conditioning or obstructive changes [9,[18], [19], [20]]. “Burnt-out” sg-cGVHD presented with inflammation-associated damage, resulting in periductal and acinar fibrosis [19,21]. Due to the similarities between SS and sg-cGVHD, studies have assessed lymphocytic foci, specifically the composite score of Imanguli et al., which involved the Chisholm & Mason score (0–4), parenchymal atrophy (0–3) and fibrosis (0–3) [8,22,23]. Wherein dysfunctional MSG, indicating “likely cGVHD”, were proposed to be associated with a Chisholm & Mason score ≥2 and a composite (Chisholm & Mason and parenchymal damage, 0–7) score ≥3, [8,22].

The sg-cGVHD infiltrate remains poorly understood but it has been discussed in comparison to SS [8]. Both disorders exhibit a strong T-cell response, and for sg-cGVHD, the markers CD4 and CD8 are reported to be increased compared to HCT-controls [9,13,20]. Macrophage (CD68), dendritic cell (CD1a) and B cell infiltration have been reported inconsistently, and as well as the comparison to SS [9,13,20]. Saliva from cGVHD patients displayed decreased salivary (s)IgA and increased IgG levels [8,24]. Increased levels of albumin, magnesium, chloride, and sodium were also reported, where sodium was proposed to be of diagnostic significance associated with MSG pathology and cGVHD [24,25].

In the current study, MSG immunopathology profiles were investigated in our large observational cohort of HCT patients [26]. Histopathological guidelines proposed by Imanguli et al., were assessed, and the NIH cGVHD Pathology Group guidelines were formalized into a grading scheme (further referred to as “NIH cGVHD grading”) [8,22,23]. As a result, we defined guided pathological diagnostics of “possible” and “likely” cGVHD [8,18,19]. In addition, MSG immune cell phenotyping with the histopathological grading was compared for disease severity [27]. We hypothesized that MSG immunopathological staging will increase our understanding of oral cGVHD pathophysiology’s [26,27].

## 2 Materials and method

### 2.1 Ethical permissions

The study was performed in accordance with the Helsinki Declaration and following permissions obtained from the Swedish Ethics Review Authority (Registration number 2013-1241-31-1, 2014/1184-31-1 and 2019-01259).

### 2.2 Study protocol and cohort

**Fig. 1:**
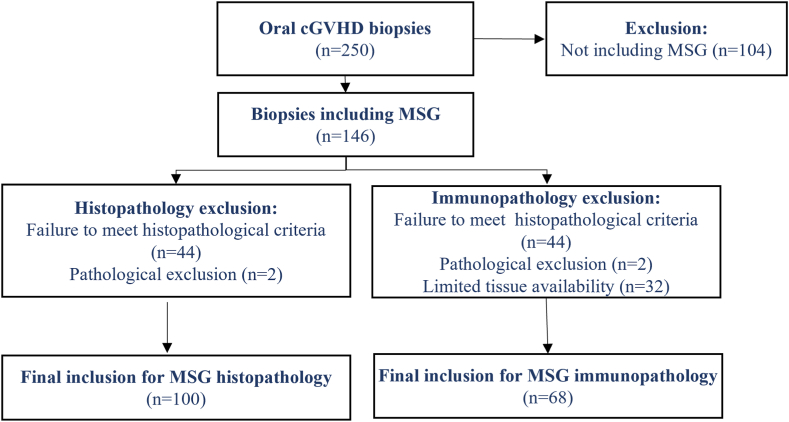
*Flow chart of study cohort biopsies.* A total of 250 oral mucosa and MSG biopsies were retrieved from the archives, of which 146 biopsies included MSG tissue. 46 were excluded due to poor quality, size or suspicion of other pathology, resulting in 100 biopsies for histological analysis. For immunopathological analysis only 68 continued to be assessed due to limited tissue availability.

The patients analyzed in this study are from a retrospective cohort of allogenic-HCT patients treated at Karolinska University Hospital (KUH) between 1977 and 2011, with biopsies retrieved from Stockholm′s Medicine Biobank (SMB) [26]. The cohort has been described previously for the studies of the om-cGVHD histopathological profile [26,27]. Biopsies had been routinely obtained as standard of care at the Oral and Maxillofacial Surgery Clinic (OMSC) KUH over the first year post-HCT at 3-month intervals, and thereafter on an individual basis for later time points (typically obtained at 3-, 6-, 9-, 12-, and ≥24-months), which in some cases resulted in more multiple biopsies per patient. 95 HCT-patients with 250 associated oral biopsies were identified from the KUH HCT-patient archive and retrieved (Fig. 1) [26]. Although, the biopsy specimens were intended for mucosal analysis, 146 biopsies from 79 patients also contained submucosal MSG tissue that were included for the purpose of histopathological investigations (Fig. 1). HCT-patients OMSC charts and KUH registry data were used to retrieve clinical information and patient characteristics (Table 1) [26]. Three healthy MSG controls were acquired from the OMSC and the Oral Medicine Public Dental Clinic at KUH, for inclusion in the histopathological assessor calibration session (details below).

**Table 1:** Clinical characteristics of HCT-patients.

Characteristics	n (% or range)
No. of patients	79
No. of MSG biopsies	146
Age and gender	
Median age (y) at HCT	31 (1–53)
Children (<18 years)	16 (20.3)
Ratio male/female	54/25 (68.4/31.6)
**Year of HCT**	
−1991	65 (82.3)
1992–2000	14 (17.7)
**Donor**	
HLA identical related	66 (83.5)
MUD	6 (7.6)
Other (Mismatched related, MRD)	7 (8.9)
Gender (unmatched/matched)	40/39 (50.6/49.4)
CMV match (pos/neg/miss/unknown	34/16/27/2 (43.0/20.3/34.2/2.5)
**Disease**	
Chronic Leukaemia	29 (36.7)
Acute Leukaemia	29 (36.7)
AA	11 (14.0)
MM	5 (6.3)
Other (Lymphoma, Metab, MF)	5 (6.3)
**Conditioning regimen**	
Myeloablative	68 (86.1)
Reduced intensity	11 (13.9)
**GVHD prophylaxis**	
CsA	15 (19.0)
MTX	14 (17.8)
CsA + MTX	43 (54.4)
TcD	5 (6.3)
None	2 (2.5)
**HCT source**	
BM**/**PBSC	74**/**5 (93.7/6.3)
**CMV infection**	
(pos/neg)	34/45 (43.0/57.0)
**aGVHD grade**	
0	23 (29.1)
1	42 (53.2)
2	14 (17.7)
**cGVHD score**	
None	24 (30.4)
Mild	38 (48.1)
Moderate	13 (16.5)
Severe	4 (5.0)

MUD – matched unrelated donor, MM - multiple myeloma, MRD – matched related donor, Metab – metabolic disorders, MF – myelofibrosis, CsA – cyclosporine A, MTX – methotrexate, TcD – T-cell depletion, BM – bone marrow, PBSC – peripheral blood stem cell.

### 2.3 Histological and immunohistochemical analyses

Original hematoxylin and eosin (H&E) stained slides were available for some SMB biopsies, and these were directly digitalized using a 3D Histech Scanner (Histolab Products AB) (detailed below). For those without original pathology slides or of poor quality, paraffin-blocks were retrieved, and samples sectioned and stained. 4 μm-thin paraffin-sections were mounted on Super Frost Plus slides (Menzel-Gläser, Braunschweig, Germany). Slides were stained with H&E (Histolab Products AB, Gothenberg, Sweden) for routine pathology, as well as PAS (Merck KGaA, Germany) for mucopolysaccharides and van Gieson (Sigma-Aldrich, Germany) for fibrosis according to the manufacturer’s instructions (Malkusch et al., 1995). Immunohistochemistry (IHC) was performed using the monoclonal antibodies, CD1a (1:15,000), CD5 (1:300), CD8 (1:1000), CD19 (1:150), CD20 (1:400) and CD68 (1:30,000) (DAKO)) according to the protocol described in Tollemar et al., 2022 [27]. All glass slides were digitalized at 40x magnification using 3D Histech Scanner and viewed in CaseViewer (Histolab Products AB). Inclusion criteria for histopathological screening of at least five lobules or an area ≥1 mm^2^ was used [8,18]. MSG tissue regions representing were identified in the digitalized images and exported from CaseViewer for analysis independent of any surrounding tissue.

### 2.4 Histopathological grading of MSG tissue in cGVHD

Histological MSG evaluation was based on the NIH cGVHD Pathology resource document (“NIH cGVHD grading”) with associated NIH specific criteria for sg-cGVHD (Table 2) [18,19]. In addition, a pathological score proposed by Imanguli et al. was also employed (Table 3) [8]. The NIH cGVHD grading form was designed to cover degree of peri-ductal and acinar infiltration, including exocytosis, ductal damage, and acinar degeneration, as well as peri-ductal and interstitial fibrosis. Each feature was assessed as mild or marked with a final pathological scoring range, and points for the features were allocated (0–16 points) (Table 2). The Imanguli et al. scoring involved a Chisholm & Mason score (0–4 points), and area of parenchymal atrophy and fibrosis scores (respectively 0–3 points), resulting with a final pathology score between 0 and 10 points (Table 3) [8,22]. The inflammatory pattern was assessed as lymphocytic, chronic-mixed or plasmocytic [9,18,19]. The highest focal score of 12 was considered as confluent inflammation [23]. Completely degenerated acini structures with inflammatory infiltrate were considered as marked migration.

**Table 2:** Histological NIH cGVHD grading for defining features of sg-cGVHD with awarded points (adapted from the NIH cGVHD Consensus Pathology Working Group resource document [16,18[16], [18]). Assessment of MSG 103 biopsies that included 100 biopsies from HCT patients and three healthy control biopsies.

Type of inflammation				
**Feature**	a) None	b) Lymphocytic	c) Plasmocytic	d) Chronic mixed
n (%)	12 (11.7)	8 (7.7)	12 (11.7)	71 (68.9)
**Ducts**				
**Features and points**	**None = 0**	**Mild = 1**	**Marked = 2**	
**1. Periductal infiltrate**	Sporadic	Focal	Widespread	
n (%)	17 (16.5)	53 (51.5)	33 (32.0)	
**2. Periductal exocytosis**	None	Focal	Widespread	
n (%)	58 (56.3)	31 (3s0.1)	14 (13.6)	
3. Ductal damage*	None	Focal	Widespread	
n (%)	57 (55.3)	31 (30.1)	15 (14.6)	
**4. Periductal fibroplasia**	Discrete	Some	Intense	
n (%)	29 (28.2)	68 (66.0)	6 (5.8)	
**Acini**				
**Features and points**	**None = 0**	**Mild = 1**	**Marked = 2**	
**5. Peri-acinar infiltrate**	Sporadic	Focal	Widespread	
n (%)	18 (17.5)	57 (55.3)	28 (27.2)	
**6. Acinar exocytosis**	None	Focal	Widespread	
n (%)	73 (70.9)	28 (27.2)	2 (1.9)	
7. Acinar destruction**	None	Focal	Widespread	
n (%)	28 (27.2)	45 (43.7)	30 (29.1)	
**8. Interstitial fibrosis**	None	Some	Intense	
n (%)	22 (21.4)	60 (58.2)	21 (20.4)	
**Total points: 16** (Grade 0 - 0–2; Grade I - 3–4; Grade II - 5–7; Grade III - 8–11; Grade IV - 12–16)				
G0; n = 13 (12.6%) Median points: 1	GI; n = 28 (27.2%) Median points: 4	GII; n = 24 (23.3%) Median points: 5	GIII; n = 21 (20.4%) Median points: 10	GIV; n = 17 (16.5%) Median points: 13

*Damage represents vacuoler changes and apoptosis. **Destruction represents atrophy, ductal metaplasia and apoptosis.

**Table 3:** Histopathological Imanguli score for defining features of sg-GVHD with score (adapted from 8). Assessment of MSG 103 biopsies that included 100 biopsies from HCT patients and three healthy control biopsies.

Chisholm & Mason					
**Features and points**	**0**	**1**	**2**	**3**	**4**
**1. Degree of infiltration**	None	Slight	Moderate or < one focus/4mm2	One focus/4mm2	>One focus/4mm2
n (%)	9 (8.7)	27 (26.2)	19 (18.45)	19 (18.45)	29 (28.2)
**Parenchymal damage**					
**Features and points**	**0**	**1**	**2**	**3**	
**2. Atrophy**	None	<33% of area	33–66% of area	>66% of area	
n (%)	18 (17.5)	45 (43.7)	22 (21.3)	18 (17.5)	
**3. Fibrosis**	None	<33% of area	33–66% of area	>66% of area	
n (%)	13 (12.6)	45 (43.7)	31 (30.1)	14 (13.6)	
**Total Imanguli points: 10** (Severity score 0 – 0-2 points; Severity score 1 – 3-6 points; Severity score 2 – 7-10 points)					
Severity score 0; n = 13 (12.6%) Median pathology points: 1		Severity score 1; n = 57 (55.4%) Median pathology points: 4		Severity score 2; n = 33 (32.0%) Median pathology points: 8	

The initial quality assessment of the exported MSG tissue was performed by an observer not involved in any grading process. A training data set of randomly selected biopsies post-HCT were selected by the same observer, which included 28 MSG biopsies and three healthy biopsies [26]. Four histopathological assessors screened the training data for re-occurring features according to the NIH cGVHD and Imanguli grading protocols [8,18,19]. Subsequent independent grading of the complete cohort was performed by the histopathological assessors and any differences were re-graded in consensus.

### 2.5 Quantitative immunohistochemistry

Quantitative-IHC used CellProfiler version 3.1.9 (www.cellprofiler.org) and pipelines were established for each antibody [[27], [28], [29]]. Images were segmented into 1000 × 1000 pixel tiles (>15 kb) using ImageMagick version 7.0.8 (www.imagemagick.org). IHC DAB chromogenic-positive staining was identified using threshold algorithms in CellProfiler. Workflows are available upon request or from https://cellprofiler.org/published-pipelines. Images with folds and artefacts were excluded. The remaining tiles were recombined to give total stained pixel area/total pixel area, and the average determined per biopsy for each antibody [[27], [28], [29]].

### 2.6 Statistics and robustness testing

Cohens weighted Kappa (κ) was performed to test the agreement by chance of the histopathological assessors grading, the two MSG grading methods and points allocation (NIH cGVHD grading and Imanguli score) [8,18,19,30]. Histopathological grading concordance was also employed for the histological assessors, grading methods using Spearman’s correlation in Prism 9 (GraphPad Software, La Jolla, CA) with a p-value ≤0.05 considered significant. For patients with repeated biopsies in the same clinical group, a mean value was plotted and used for the correlation coefficient. Cluster analysis of the training data pathology points were used to group the NIH cGVHD grading into more defined groups of G0 to GIV, and for Imanguli Score 0–2. In addition, Jenks natural breaks optimization for one dimensional data was used to define boundary values and to confirm the classification of the data into groups [31,32].

Evaluation of the quantitative-IHC results were performed using generalized estimating equations with an independent working correlation matrix that considered potential intrasubject correlation when estimating standard errors [27,28,33]. A gamma distribution family with log-link was adopted, as pixel area showed a positive and skewed distribution in each group. Group-averaged pixel area was compared across the NIH cGVHD grading (G0-GIV) and Imanguli scores (score 0–2). Results for the immunopathology MSG analysis were reported as marginally predicted fold-change (mean pixel area and mean pixel area ratios) using G0-I (NIH cGVHD grade) or score 0 (Imanguli score) as reference [27]. Pairwise comparisons were conducted between the different grades/scores in the two separate grading schemes. p-values of <0.05 were considered as significant and 95% confidence intervals (CI) are given in the Supplementary Information (S). Analyses were performed with Stata version 16 (StataCorp, College Station, TX).

## 3 Results

### 3.1 Patient characteristics and biopsy cohort

The majority of the cohort had undergone conventional HCT-techniques with myeloablative conditioning (MAC) and the use of bone marrow stem cells (Table 1). Seven patients that received MAC had chemotherapy solely involving busulfan and cyclophosphamide, whereas the others had combinations of fractioned- (n = 3) or total- (n = 60) irradiation with chemotherapy. Global acute and cGVHD were mainly mild. Twelve patients developed cGVHD de novo, whereas 44 suffered with previous aGVHD. The median day of cGVHD onset was at day 144. Symptoms of mouth dryness were included within the archived patient charts, however as limitation to the current study, the reporting in these journals was not standardized and was therefore not analyzed further in our cohort.

Of the 146 MSG biopsies (n = 79 patients) obtained from the archive, 102 HCT-biopsies met the inclusion criteria for histopathological analysis, but two biopsies were additionally excluded; one showed completely degenerated and fibrotic parenchyma which prevented the assessment of the tissue pathology, and the other was diagnosed with a mucous extravasation cyst (Fig. 1). Biopsies were from patients prior HCT (n = 10), with diagnostic and/or distinctive features of oral cGVHD (n = 53) or from patients without oral cGVHD (n = 37). Immunohistochemical MSG infiltrate with associated pathology grades was assessed on 68 MSG biopsies, since 32 biopsies were excluded due to too small tissue size or limited tissue availability (Fig. 1). Antibody specific biopsy exclusion was due to poor quality post staining and quantification (CD4 n = 2, CD8 n = 4, CD68 n = 2 and CD1a n = 5).

### 3.2 Validation of histopathological assessment

The primary infiltrate was lymphocytic, but mixed chronic mononuclear cells were consistently present. Jenks natural breaks was used to define the separation of the pathology severity, with the NIH cGVHD grading as G0: 0–2 points, GI: 3–4 points, GII: 5–7 points, GIII: 8–11 points and GIV:12–16 points, and severity scores for Imanguli as Score 0: 0–2 points, Score 1: 3–6 points and Score 2: 7–10 points respectively (Table 2, Table 3, S1). Histopathological calibration concordance displayed strong agreement (p < 0.0001) for the NIH cGVHD grading (0–16 points) (r = 0.79), and Imanguli score (0–10 points) (r = 0.83) between the assessors. Larger blood vessels surrounding the MSG, as well as the excretory ducts, displayed varying degrees of inflammation but were not included within the MSG criteria [18,19].

### 3.3 Full cohort histopathological analysis using the NIH cGVHD grading scheme

**Fig. 2:**
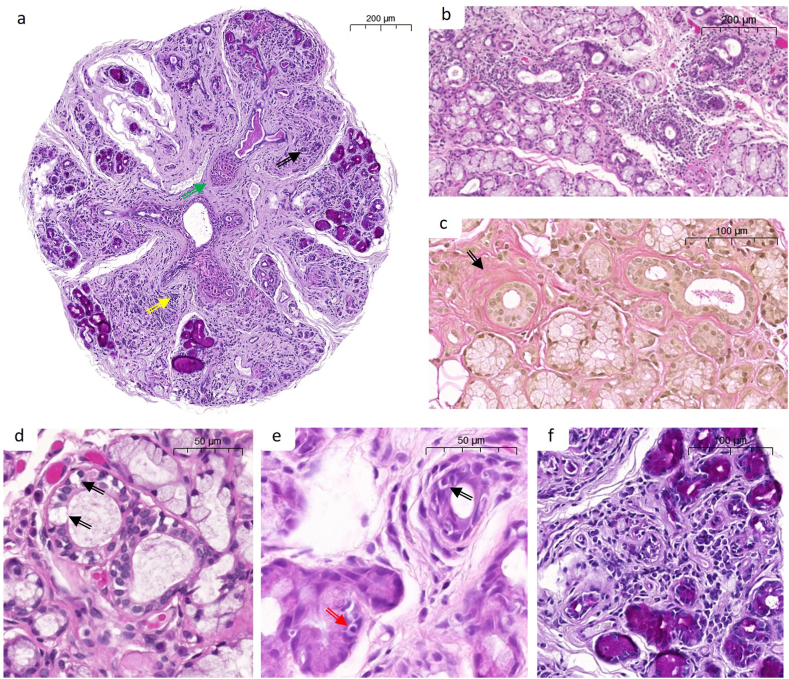
*Representative images of the more severe histopathological features found in MSG of cGVHD patients.* a. MSG stained with PAS staining demonstrating acinar destruction (black arrow), widespread mononuclear infiltrate (see f. yellow arrow), and fibrosis (green arrow). b. H&E-stained tissue showing periductal mononuclear infiltrate around the central ducts, c. Periductal fibrosis demonstrated by van Gieson’s staining (black arrow). d. Damage to intralobular duct in the form of vacuolization (black arrow). e. Exocytosis into intralobular duct (black arrow), as well acinar exocytosis (red arrow). f. Higher magnification of periacinar mononuclear infiltrate. Size bars: a, b, and c - 200 μm, d and e − 50 μm, and f - 100 μm.

Full cohort evaluation showed wide distribution of histopathological features and severity grades (Fig. 2 a-f and Table 2). Twelve biopsies displayed no inflammation whereas the others were considered; lymphocytic (8; 7.7%), plasmocytic (12; 11.7%) or chronic mixed (71; 68.9%). The inflammatory severity infiltrate showed similar distribution between ducts and acini, whereas marked exocytosis was more commonly found within the ductal epithelium (Fig. 2 a, b, e-f and Table 2). Acinar degeneration and interstitial fibrosis were more severe than ductal damage and periductal fibrosis (Fig. 2 a, c-d, f). Based upon the NIH specific criteria for active GVHD pathology, compiled points of ≥2 for ductal and/or acinar inflammation, as well as ≥2 for ductal damage and/or acinar degeneration, were considered as active disease. Samples meeting those criteria involved all GIII-GIV (n = 38; 100%, median points 11) and four biopsies with the grade of GII (16.7%, median points 6.5). None of the biopsies meeting the NIH specific criteria were of plasmocytic infiltrate. Therefore, we propose, in similarity to the grading of om-cGVHD; G0-GI for no/inactive pathology, GII for diagnostic “possible” and GIII-GIV for diagnostic “likely” cGVHD [19,26,27].

### 3.4 Full cohort histopathological analysis using the imanguli scoring

The Imanguli score exhibited large variations across the cohort (Fig. 2, Table 3). The degree of infiltration was widely distributed between the Chisholm & Mason; 1 (27; 26.2%), 2 (19; 18.45%), 3 (19; 18.45%) and 4 (29; 28.2%) (Table 3) [22], whereas atrophy and fibrosis for parenchymal damage were found to be <33% (point 1; 45; 43.7%) (Table 3). Correlation of atrophy and fibrosis feature points showed a relatively strong agreement (r = 0.70, p < 0.0001). Diagnostic “likely” cGVHD was suggested as lymphocytic Chisholm & Mason of ≥2 with additional features of ≥1 using combined atrophy and fibrosis. 54 biopsies fulfilled the criteria: 21 (37.5%) biopsies originally of Score 1 (median pathology points 6) and 33 (100%) of Score 2 (median pathology points 8). In summary, the Imanguli scoring was designated as diagnostic; Score 1 for “possible” and Score 2 for “likely” cGVHD.

A strong correlation (r = 0.90, p < 0.0001) was found between the final pathology points between the NIH cGVHD grading (0–16 points) and the Imanguli scoring (0–10 points) (S2). More specifically, the agreement between G0-GI and Score 0, GII and Score 1, and GIII-GIV and Score 2 between the two grading schemes were moderate to substantial (κ 0.61) (S2).

### 3.5 Immunohistopathological characterization of glandular infiltrate

**Fig. 3:**
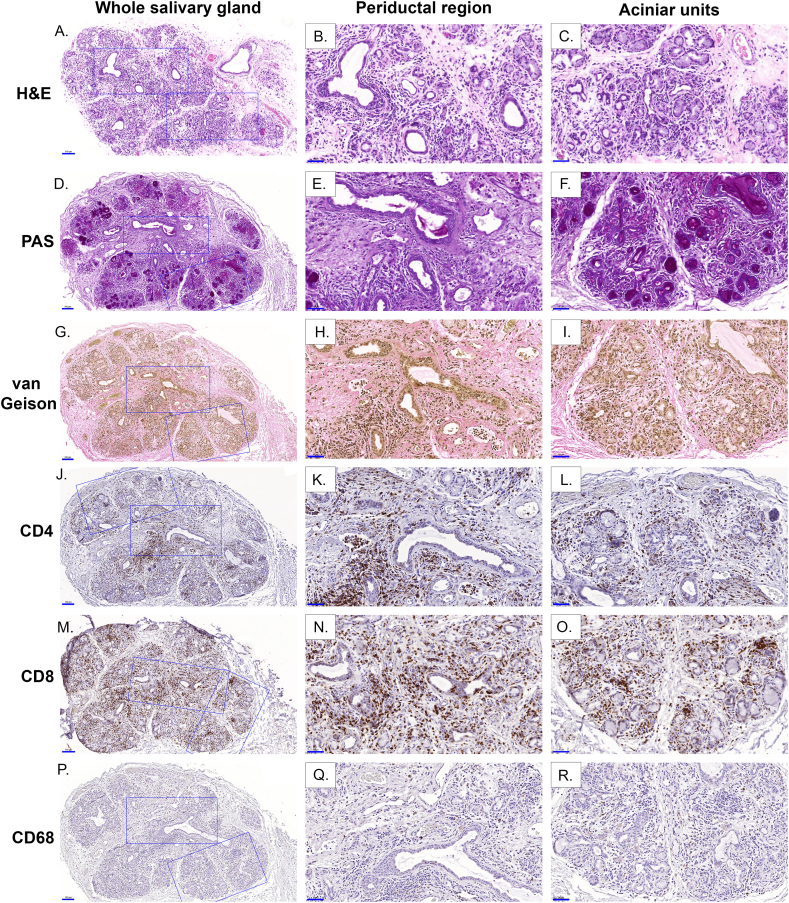
*Histological features and immunohistochemical localization of immune cell markers in MSG cGVHD.* The image shows a MSG biopsy taken 195 days post-HCT and graded using the NIH cGVHD grading with GIV (14 points) and the Imanguli Score 2 (10 points). H&E (A–C) showed the overall pathology and was complemented with PAS (D–F) and van Gieson (G–I) for visualization of acinar destruction and fibrosis respectively. Immunohistochemical staining is shown for the antibodies; CD4 (J–L), CD8 (M − O) and CD68 (P–R) but not CD1a (not shown) as no positive stain was detected. Whole salivary gland (A, D, G, J, M and P) at higher magnification (size bar = 100 μm) and the boxes denote the magnified periductal regions (B, E, H, K, N and Q) and acinar units (C, F, I, L, O and R) (size bar 50 μm).

T-cell infiltration (CD4 and CD8) was found as the predominant cell type (Fig. 3 A - 0). T-cell immunolocalization was observed around ducts and acini, and with increased severity these cells migrated into the epithelial/acinar cells (Fig. 3 J – O). Macrophages (CD68) were found around ducts and within the interstitial and acinar areas of most biopsies, although this varied between individuals (Fig. 3 P - R). Dendritic cells (CD1a) were sparsely found in MSG, compared to the oral mucosa (positive control), despite some minor staining with a few patients (images not shown). B-cell (CD19 and CD20) localization in our cohort was found to be rare if any present (data not shown), and therefore not analyzed further in this study.

### 3.6 Immune cell characterization and association to histopathological grades

**Fig. 4:**
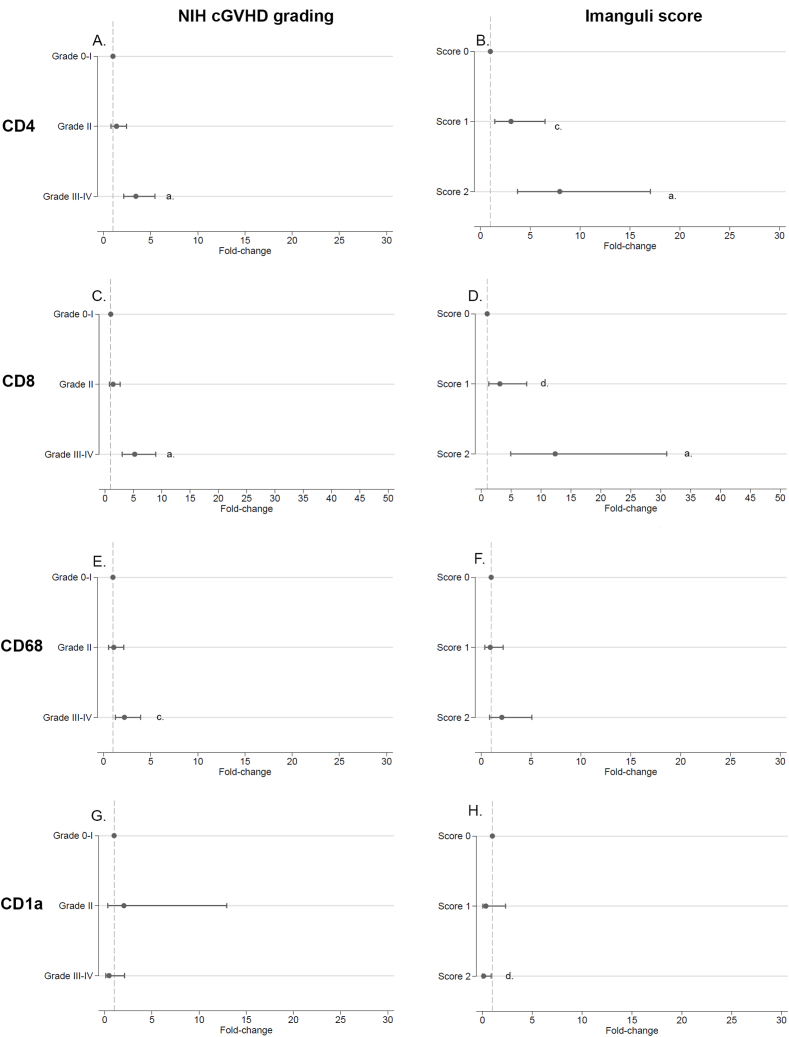
*Quantification of immune phenotypic marker localization with respect to histological grading system in MSG cGVHD.* Quantification of immunohistochemical (CD4 (A and B), CD8 (C and D), CD68 (E and F) and CD1a (G and H)) staining was performed using generalized estimating equations with a gamma distribution. Comparisons are shown with fold-change between different histological grading schemes (NIH cGVHD grading and Imanguli score) and severities. NIH cGVHD grading was divided into G0- GI for inconsistent with sg-cGVHD, GII “possible sg-cGVHD”, and GIII-GIV “likely sg-cGVHD”. Imanguli scores were divided into score 0 inconsistent with sg-cGVHD, Score 1 “possible sg-cGVHD”, and Score 2 “likely cGVHD”. The dotted line indicates the normalized value [1] for G0 or Score 0 respectively. P values a = ≤ 0.001, b = ≤ 0.005, c = ≤0.01 and d = ≤ 0.05. Detailed numbers pertaining to significance can be found in S3 along with pairwise analyses between the groups.

Quantification of the immune cell profile with pathological grading found CD4 and CD8 to be elevated with severity (Fig. 4 A - D). Diagnostic “likely cGVHD” (NIH cGVHD GIII-GIV) biopsies were found with increased CD4 (3-fold p < 0.001) and CD8 (5-fold p < 0.001) compared to NIH cGVHD (G0-GI). The Imanguli Score 2 representing diagnostic “likely cGVHD” was also significantly increased, with CD4 (8-fold p < 0.001) and CD8 (12-fold p < 0.001) compared to Score 0 (Fig. 4 A - D, S3). CD68 was only significant for NIH cGVHD (GIII-GIV) (2-fold, p < 0.01) compared to G0-GI (Fig. 4 E − F, S3).

## 4 Discussion

Salivary gland-cGVHD and HCT-related mouth dryness remains poorly understood and lacks diagnostic criteria. Studies have pointed to distinctions between sg-cGVHD and om-cGVHD [5,8,11,34]. Herein, we assessed the NIH cGVHD grading using the histopathological resource document for specific sg-cGVHD pathology criteria, in parallel to validating the Imanguli scoring, and assessing well-described immune profiles of cGVHD pathogenesis [8,18,19]. The histological grading across our large cohort of HCT patients and biopsies is one of the most extensive studies of MSG histopathology post allogenic-HCT.

Recurring histological features were defined in the NIH cGVHD grading scheme of periductal inflammation and fibrosis, acinar inflammation and interstitial fibrosis, lymphocytic ductal and acinar exocytosis with damage/degeneration to ducts and acini [18,19]. Soares and co-authors reported peri-ductal lymphocytic infiltrate and exocytosis as the principal feature contributing to decreased overall survival in cGVHD patients [9]. Hypothetically they postulated it was a sign of disease activity with subsequent parenchymal loss, resulting in co-morbidities as xerostomia, oral infections and decreased food intake and quality of life. In the current investigation we addressed the need to determine the magnitude and location of the lymphocytic inflammation [19]. Biopsies meeting the criteria of “likely cGVHD” were mostly defined with marked peri-ductal/acinar infiltrate according to the NIH cGVHD grading. Exocytosis was predominately found in the ducts [9]. However, some samples displayed focal infiltrate but consistent exocytosis and were therefore defined as “likely cGVHD”. It remains to be addressed whether there is a need to define apoptosis when lymphocytic exocytosis is present [19]. Apoptosis was not included as a separate feature, but along with vacuolization and atrophy considered a hallmark for damage/degeneration. Fibroplasia was also considered as a specific feature but not necessarily associated with cGVHD activity [18,19]. Ductal ectasia and rupture could indicate non-specific salivary gland damage and are reported to not correlate to cGVHD [8,9,[18], [19], [20]]. Additionally, oncocytic metaplasia, a potential feature in pediatric GVHD, was not assessed as our cohort displayed broad differences in age [18,19]. Consequently, active glandular NIH cGVHD grading specifically included lymphocytic (or mixed) ductal and acinar inflammation, exocytosis, and cell damage/degeneration, which we denoted pathologically diagnostic of “possible sg-cGVHD” at GII and “likely sg-cGVHD” at GIII-GIV [18,19].

The clinical resemblance between sg-cGVHD and SS has been reported, whereas the histopathological features might differ [11,35,36]. The Imanguli scoring included inflammatory focus, and combined fibrosis and atrophy [8]. In our cohort, inflammatory infiltrate was diffuse, although extensive in many cases, contrary to the typical focus score seen in SS [36,37]. However, when present, the scoring frequently corresponded to a diagnosis of “likely cGVHD”. We assessed the Imanguli score with atrophy and fibrosis separately, which enabled us to derive diagnostic scores for both “possible (Score 1)” and likely (Score 2)” cGVHD. Differences in the severity of atrophy and fibrosis have been reported by others and we found a relatively strong correlation, which was in line with that reported by Imanguli and colleagues [8,20].

Overall, the NIH cGVHD grading and Imanguli scoring showed good agreement for pathology score, but some discrepancies were noted for diagnostic grades of “possible or likely”. Imanguli score was found with a slightly higher concordance between the assessors, which could be attributed to the simplified criteria. However, the more specific NIH cGVHD grading criteria might be better suited for small tissue biopsies and research purposes to determine “possible and likely” specificity and facilitating in depth evaluation of for instance exocytosis and apoptotic damage. Ideally 5–10 surgically removed individual MGS are recommended for reliable diagnostics but unfortunately considering the current retrospective cohort it was not possible to fully comply [14,18,19]. Furthermore, evidence does suggest that not all glandular segments from the same biopsy are involved in the disease, and that completely fibrotic tissue segments only suggest previous activity [18,19]. However, to date, most research performed on sg-cGVHD has examined mucosal and glandular biopsies, in line with the current approach [9,13,20,36].

The primary infiltrate in the current cohort was typically lymphocytic but plasma cell involvement was found throughout the patient samples [9]. To date, few studies have explored the immunopathological profile in sg-cGVHD. Strong infiltrates of CD4 and CD8 cells, were found to increase with pathological severity, especially for CD8 [9,13,20,36]. CD68 immunolocalization showed slight rises within the MSGs, that increased with pathological severity. However, there is limited knowledge regarding the role and extent of macrophage localization in sg-cGVHD, which warrants further investigation [9,20]. Of note, all study comparisons were made against the HCT-biopsies G0-GI. We observed patient-dependent variations in CD68 localization, but intra-individual comparisons showed lower CD68 in the MSG compared to the oral mucosa. Both dendritic cells (CD1a) and B-cells (CD20) were found to be rare cell types in the MSG, in contrast to SS pathobiology, where antigen presenting cells and B-cells are commonly observed, and CD4 predominate the T-cell infiltrate [13,20,36,37]. It was out of the scope of the current study to perform comparative histopathological analyses against SS-patient tissues, but the need remains to perform such investigations to fully establish any links between the two disorders.

In conclusion, we validated sg-cGVHD pathological scoring, and based upon the NIH specific criteria we propose severity grading for sg-cGVHD [8,18,19]. Therefore, to ensure a clinical classification of distinctive sg-cGVHD, pathological confirmation of “likely cGVHD” is required [5,19]. Studies have suggested that low saliva rates continue in cGVHD patients, which could also be used as a late clinical confirmation [12,35,38,39]. We acknowledge that salivary gland damage might be due to the conditioning regimes, unspecific trauma, and a response of overall cGVHD, which is difficult to determine but it highlights the need for large cohort defined clinicopathological studies. Thus, it is crucial to identify early diagnostic criteria for sg-cGVHD and saliva biomarkers could potentially serve this purpose in the future [40,41].

## Funding

Financial support from Styrgruppen KI/Region Stockholm för Odontologisk Forskning, ALF Medicine Region Stockholm, Swedish Dental Society, Swedish Society for Orofacial Medicine and Karolinska Institutet.

## Author contribution statement

Victor Tollemar; Rachael Sugars: Conceived and designed the experiments; Performed the experiments; Analyzed and interpreted the data; Contributed reagents, materials, analysis tools or data; Wrote the paper.

Helena Arvidsson; Nikolce Tudzarovski: Performed the experiments; Contributed reagents, materials, analysis tools or data; Wrote the paper.

Henrike Häbel: Analyzed and interpreted the data; Contributed reagents, materials, analysis tools or data; Wrote the paper.

Karin Garming Legert: Performed the experiments; Wrote the paper.

Katarina Le Blanc: Conceived and designed the experiments; Wrote the paper.

Gunnar Warfvinge: Conceived and designed the experiments; Performed the experiments; Contributed reagents, materials, analysis tools or data; Wrote the paper.

## Data availability statement

Data will be made available on request.

## Declaration of interest’s statement

The authors declare no conflict of interest.
