# Supplementary material for: Grading of minor salivary gland immuno-histopathology post-allogenic hematopoietic cell transplantation
Source: Heliyon. 2023 Apr 15;9(4):e15517. doi: 10.1016/j.heliyon.2023.e15517 (PMC10148098; doi:10.1016/j.heliyon.2023.e15517)
Supplement: Multimedia component 1 [file mmc1.docx]

Minor Salivary Gland Immuno-Histopathology in Chronic Graft-versus-Host Disease

V. Tollemar, H. Arvidsson, H. Häbel, N. Tudzarovski, K. Garming Legert, K. Le Blanc, G. Warfvinge, and R.V. Sugars.

Supplementary Material

**Supplementary Figure 1 (S1)** *Grading thresholds by cluster analysis for the histological grading schemes for MSG.* A validation cohort (n=28 biopsies) was used to determine histological grading thresholds of MSG. One biopsy was excluded since consensus could not be reached amongst the assessors. Two scoring methods were used; the NIH cGVHD grading (total 16 points) (<https://www.astct.org/archive/practice-resources/nih-chronic-gvhd-consensus-project>), and the Imanguli scoring (total of 10 points) (Imanguli et al., 2010). Points were allocated for the respective features (see Tables 2 & 3). To define the classification range and associated histological grade, the output of each scoring system was clustered using Jenks natural breaks optimization. NIH cGVHD grading was divided into Grades 0-IV (G0-GIV). G0 (0-2points) and GI (3-4 points) were both considered inconsistent with sg-cGVHD, GII (5-7 points) possible sg-cGVHD, and GIII (8-11 points) and GIV (12-16 points) both likely cGVHD. Imanguli scores were divided into Scores 0-2, where score 0 (0-2 points) was considered inconsistent with sg-cGVHD, score 1 (3-6 points) possible sg-cGVHD, and score 2 (7-10 points) likely cGVHD.


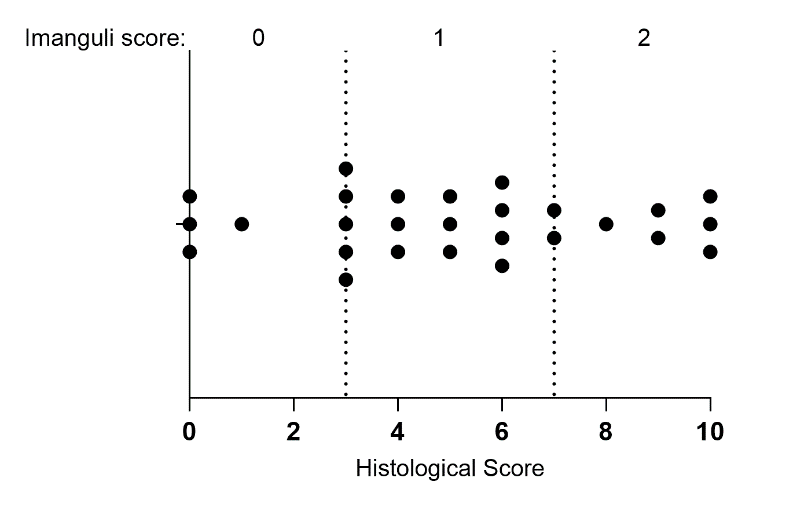

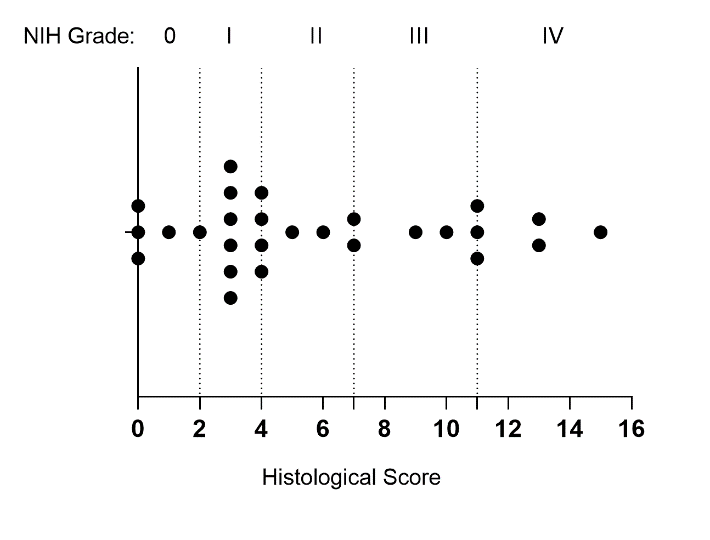


**Supplementary Figure 2 (S2).**
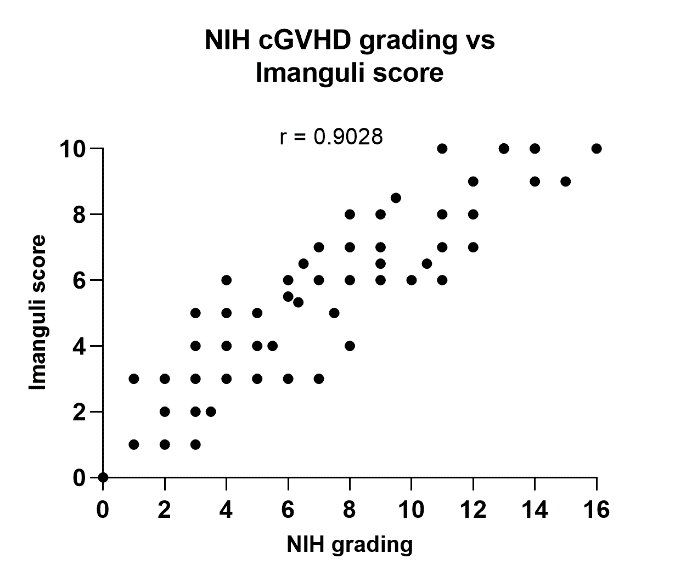
*Concordance between the NIH cGVHD grading and the Imanguli score was assessed across the whole cohort*. Spearman’s correlation was used to determine the r score, and excellent agreement was found between the two grading schemes for MSG cGVHD. The agreement for diagnostic groups were substanial, determined by the weighted kappa (κ) .

|  |  | | Weighted kappa (κ): 0.61 | | |
| --- | --- | --- | --- | --- | --- |
|  | *GIII-GIV* | | 0 | 6 | 32 |
| NIH  cGVHD  grade | *GII* | | 0 | 23 | 1 |
|  | *G0-GI* | | 13 | 28 | 0 |
|  |  | | *Score 0* | *Score 1* | *Score 2* |
|  |  | Imanguli score | | | |

**Supplementary Figure 3 (S3).** *Quantification of immune cell marker localization by image analysis based upon NIH cGVHD grading (G0-IV) and Imanguli score (0-2) for sg-GVHD.* Quantification of immunohistochemical (CD4, CD8, CD1a and CD68) staining was performed using generalized estimating equations with a gamma distribution and log-link and demonstrated significant changes in mean pixel area ratio (fold-change) between different histopathological grades. Tables show the pixel-area fold-changes with 95% confidence intervals with respect to the histopathological grade. All analyses presented in the table are compared against G0 (NIH) or Score 0 (Imanguli), which is set at 1.

P values a=≤ 0.001, b=≤ 0.005, c=≤0.01 and d=≤ 0.05.

**CD4 – NIH grade**

Number of obs (NoB) = 66

------------------------------------------------------------------------------------------
 CD4 MSG | Fold-change Std. Err. z P>|z| [95% Conf. Interval]
 --------------+----------------------------------------------------------------------------
 Grade 0- I |
 Grade II | 1.384 0.400 1.13 0.260 0.786 2.437
 Grade III-IV | 3.424 0.811 5.19 0.000 2.152 5.448 a.
 |
 _cons | 0.004 0.001 -31.16 0.000 0.003 0.005
 -----------------------------------------------------------------------------------------

**NoB Fold-change p-value**

Grade II vs Grade III-IV 44 2.474 0.001 a.

**CD4 – Imanguli score**

NoB = 66

--------------------------------------------------------------------------------------------
 CD4 MSG | Fold-change Std. Err. z P>|z| [95% Conf. Interval]
 ----------------+---------------------------------------------------------------------------
 Score 0 |
 Score 1 | 3.072 1.172 2.94 0.003 1.455 6.489 c.
 Score 2 | 7.964 3.090 5.35 0.000 3.723 17.035 a.
 |
 _cons | 0.00 0.001 -18.28 0.000 0.001 0.003
 --------------------------------------------------------------------------------------------

**NoB Fold-change p-value**

Score 1 vs Score 2 60 2.593 0.000 a.

**CD8 – NIH grade**

NoB = 64

--------------------------------------------------------------------------------------------
 CD8 MSG | Fold-change Std. Err. z P>|z| [95% Conf. Interval]
 ------------- ---+-------------------------------------------------------------------------
 Grade 0-I |
 Grade II | 1.412 0.452 1.08 0.281 0.754 2.650
 Grade III-IV | 5.219 1.434 6.01 0.000 3.046 8.944 a.
 |
 _cons | 0.005 0.001 -26.14 0.000 0.003 0.007
 -------------------------------------------------------------------------------------------

**NoB Fold-change p-value**

Grade II vs Grade III-IV 42 3.697 0.000 a.

**CD8 – Imanguli score**

NoB = 64

---------------------------------------------------------------------------------------------
 CD8 MSG | Fold-change Std. Err. z P>|z| [95% Conf. Interval]
 ----------------+---------------------------------------------------------------------------
 Score 0 |
 Score 1 | 3.126 1.423 2.50 0.012 1.280 7.631 d.
 Score 2 | 12.379 5.796 5.37 0.000 4.944 30.992 a.
 |
 _cons | 0.002 0.001 -14.40 0.000 0.001 0.005
 ---------------------------------------------------------------------------------------------

**NoB Fold-change p-value**

Score 1 vs Score 2 59 3.960 0.000 a.

**CD68 – NIH grade**

NoB = 66

----------------------------------------------------------------------------------------------
 CD68 MSG | Fold-change Std. Err. z P>|z| [95% Conf. Interval]
 ----------------+--------------------------------------------------------------------------
 Grade 0-I |
 Grade II | 1.090 0.376 0.25 0.804 0.554 2.143
 Grade III-IV | 2.219 0.647 2.73 0.006 1.253 3.930 c.
 |
 _cons | 0.002 0.001 -27.59 0.000 0.002 0.004
 ---------------------------------------------------------------------------------------------

**NoB Fold-change p-value**

Grade II vs Grade III-IV 44 2.037 0.026 d.

**CD68 – Imanguli score**

NoB = 66

--------------------------------------------------------------------------------------------
 CD68 MSG | Fold-change Std. Err. z P>|z| [95% Conf. Interval]
 ----------------+--------------------------------------------------------------------------
 Score 0 |
 Score 1 | 0.908 0.408 -0.21 0.830 0.376 2.191
 Score 2 | 2.072 0.952 1.59 0.113 0.842 5.100
 |
 _cons | 0.003 0.001 -14.35 0.000 0.001 0.006
 ---------------------------------------------------------------------------------------------

**NoB Fold-change p-value**

Score 1 vs Score 2 60 2.281 0.002 b.

**CD1a – NIH grade**

NoB = 63

-----------------------------------------------------------------------------------------------
 CD1a MSG | Fold-change Std. Err. z P>|z| [95% Conf. Interval]
 ----------------+------------------------------------------------------------------------------
 Grade 0-I |
 Grade II | 2.036 1.921 0.75 0.451 0.320 12.942
 Grade III-IV | 0.467 0.359 -0.99 0.322 0.104 2.107
 |
 _cons | 0.001 0.000 -16.38 0.000 0.000 0.000
 -----------------------------------------------------------------------------------------------

**NoB Fold-change p-value**

Grade II vs Grade III-IV 41 0.230 0.032 d.

**CD1a – Imanguli score**

NoB = 63

---------------------------------------------------------------------------------------------
 CD1a MSG | Fold-change Std. Err. z P>|z| [95% Conf. Interval]
 ----------------+----------------------------------------------------------------------------
 Score 0 |
 Score 1 | 0.350 0.338 -1.09 0.277 0.053 2.316
 Score 2 | 0.125 0.123 -2.11 0.035 0.018 0.863 d.
 |
 _cons | 0.000 0.000 -9.40 0.000 0.000 0.001
 -------------------------------------------------------------------------------------------

**NoB Fold-change p-value**

Score 1 vs Score 2 57 0.358 0.076

**Reference**

Imanguli, M. M., Atkinson, J. C., Mitchell, S. A., Avila, D. N., Bishop, R. J., Cowen, E. W., . . . Pavletic, S. Z. (2010). Salivary Gland Involvement in cGVHD: Prevalence, Clinical Significance, and Recommendations for Evaluation. *Biol Blood Marrow Transplant,* 16(10), 1362-1369.
